# Nanoparticles combined with cefixime as an effective synergistic strategy against *Salmonella enterica typhi*

**DOI:** 10.1016/j.sjbs.2021.05.032

**Published:** 2021-05-19

**Authors:** Chintan Kapadia, Alaa Alhazmi, Nafisa Patel, Basem H. Elesawy, R.Z. Sayyed, Fatema Lokhandwala, Shafiul Haque, Rahul Datta

**Affiliations:** a Navsari Agricultural University, Navsari, Gujarat, India; b Naranalala College of Professional and Applied Sciences, Navsari, Gujarat, India; c Department of Clinical Laboratory Sciences, College of Applied Medical Sciences, Taif University, P.O. Box 11099, Taif 21944, Saudi Arabia; d Dept. of Microbiology, PSGVP Mandal’s Arts, Sci & Comm College, SHAHADA-425409 (MS), India; e Medical Laboratory Technology Department, Jazan University, Jazan, Saudi Arabia; f SMIRES for Consultation in Specialized Medical Laboratories, Jazan University, Jazan, Saudi Arabia; g Research & Scientific Studies Unit, College of Nursing & Allied Health Sciences, Jazan University, Jazan 45142, Saudi Arabia; h Department of Geology and Pedology, Faculty of Forestry and Wood Technology, Mendel University in Brno, Zemedelska 1, 613 00 Brno, Czech Republic

**Keywords:** *Salmonella enterica typhi*, Ag, Ni, Cu, Zn, Synergistic, Antimicrobial

## Abstract

*Enteric fever caused by Salmonella typhi* has been the most crucial health issue in rural people, especially in Southeast Asia and Africa. *Another disease, Salmonellosis*, caused by a large group of bacteria of the genus *Salmonella*, cause substantial economic loss resulting from mortality and morbidity. Higher concentration and repeated use of antibiotics to treat these diseases will likely develop antibiotic resistance among the microbes. The nanoparticle has good penetration power and can kill microbes. Combining two strategies by using nanoparticles with antibiotics kills microbes and reduces the chances of the development of antibiotics resistance. Silver, Nickel, Copper, and Zinc oxide Nanoparticles were chemically synthesized and characterized in this study. Silver nanoparticles at a concentration of 10 µg/ml inhibit all the strains under study.

In comparison, silver nanoparticles (16.90 µg/ml), Nickel nanoparticles (83 µg ml^−1^), Copper nanoparticles (249 µg ml^−1^), and Zinc oxide (1614 µg ml^−1^) along with 50 µg/ml cefixime gave maximum zone of inhibition of 35 mm, 19 mm, 31 mm and 23 mm respectively. The antimicrobial assay showed that silver nanoparticles presented good antibacterial performance against all multi-drug-resistant pathogenic *Salmonella* sp alone as well as in combinations. The present study proved that silver nanoparticles at the lowest concentration along with cefixime could be a possible alternative to control the multi-drug-resistant pathogens.

## 1 Introduction

Salmonella subspecies enterica subtype typhi is a causative agent of Salmonellosis, a disease that appeared during poor hygiene and contaminated food or water. The disease will lead to chronic typhoid fever and bloody diarrhea (Ahmad et al., 2014). Several serotypes of salmonella typhi exist in different parts of the world and share a structural similarity. There has been some well-proven, effective remedy available for treating such disease viz. chloramphenicol, cotrimoxazole, ampicillin, amoxicillin, fluoroquinolones, cephalosporins, cefixime either alone or in combination (Chaudhary et al., 2013). However, the development of drug resistance among the pathogens has been the main limiting factor of these drugs (AL-Waili, 2012, AL-Waili, 2013, Ansari, 2013). There are reports regarding resistance against chloramphenicol during 1972. Some scientists reported a combination of azithromycin with cefixime as the best possible, safer, and responsive substitutes to a standard single drug. Bacteria gradually develop resistance towards common antibiotics as a mechanism of their survival (Al-Waili, 2012; Mather et al., 2013).

Nanoparticles being smaller molecules with a high surface area to volume ratio are preferred sources for various applications, including antimicrobials (Ghramh, 2019; Mukherjee et al., 2014). Though antibiotics exert lethal effects on pathogens, they sometimes fail to efficiently inhibit the pathogen due to their poor transport across the pathogen's cell membrane (Huh and Kwon, 2011). Moreover, the drug-resistant strains will create more hindrance in successful therapy. There has been continuous emergence of multidrug resistance pathogens (Huh and Kwon, 2011) due to uncontrollable usage of antimicrobial agents or inherent capabilities of the pathogens to combat against antibiotics. These multi-drug-resistant pathogens are life-threatening if not correctly cures or killed. This increases multidrug resistance in pathogens impose to look for effective and novel methods to cure disease. Several reports mentioned the usefulness of metal nanoparticles to control pathogens. The combination of NPs with antibiotics offers enhanced antimicrobial effects compared to single-molecule alone. This combination offers numerous advantages, such as no risk of developing resistance in the pathogen, lower cytotoxic effects, and no health risk.

The present investigations aimed to chemically synthesize different nanoparticles like Nickel, Zinc, Copper, and Silver and study their antimicrobial effects against several common strains and multidrug-resistant strains of Salmonella subspecies enterica subtype typhi. Moreover, the combinatorial effects of cefixime with different nanoparticles were evaluated against pathogens to eliminate the possible chances of developing antibiotics resistance and as a future strategy.

## 2 Materials and methods

### 2.1 Source of microbial cultures

Antibiotic-resistant Salmonella typhi, S. paratyphi A, S. paratyphi B, MDR 124, MDR STA2, and MDR AST*s* were obtained from microbial culture repository of Naranlala College of a pure and applied science laboratory, Navsari, Gujarat, India.

### 2.2 Chemical synthesis of nanoparticles

#### 2.2.1 Synthesis of silver nanoparticles

A solution consisting of 0.0169 g of AgNO_3_ in 100 ml distilled water was incubated at 90 °C for 5 min followed by adding 1.25 ml of 1% (w/v) solution of trisodium citrate. As soon as the color change began, the reaction mixture was cooled and stirred by vortexing for 15 min (Rashid et al., 2013).

#### 2.2.2 Synthesis of Nickel nanoparticle

A solution of 0.1 g of nickel chloride hexahydrate in 10 ml of distilled water was mixed with 10 ml of 0.4 N NaOH and 10 ml hydrazine hydrate. For stabilization of nanoparticle, 0.2 g Polyvinyl pyrrolidone (PVP) in 3 ml of ethanol was added in solution B. Solution B was added to solution A. This resulted in a blue color solution incubated in a microwave oven for 5 min (Rose et al., 2017).

#### 2.2.3 Synthesis of Zinc oxide nanoparticles

A 9.68 g of zinc sulfate was dissolved in 120 ml of distilled water, then 10 ml of 0.1 N NaOH solution was slowly added with continuous stirring and incubated for 24 h followed by paper filtration. The pellet was dried at 150 °C for 5 h and then cool (Kumar and Karimi, 2015).

#### 2.2.4 Synthesis of copper nanoparticles

For this, in 0.1 M copper (II) sulfate pentahydrate solution, 50 ml of 0.2 M ascorbic acid solution was added, and the solution was continuously stirred. To this, 30 ml of 1 M NaOH solution was added, and the solution was heated at 80 °C for 2 h and observed for the color change to yellow (Khan et al., 2016).

### 2.3 Characterization of Ag NPs

#### 2.3.1 U.V.–Vis spectroscopy

Smaller particle tends to absorb light at different wavelengths than their salt. The several nanoparticles mentioned above were analyzed under Shimadzu U.V./vis 2600 spectrophotometer and measured absorption maxima between 185 and 1000 nm.

#### 2.3.2 Fourier transform infrared spectroscopy (FTIR)

The nanoparticles were prepared by the potassium bromide method and diluted with a 1:99 ratio of sample to potassium bromide. The spectra were scanned between 400 and 4000 cm^−1^ in FTIR (Chattopadhyay et al., 2012) equipped with a (PerkinElmer Spectrum RX I system).

#### 2.3.3 Scanning electron microscopy (SEM)

A one mg ml^−1^ concentration of each metal NPs was separately dissolved in deionized water and sonicated to get a homogeneous solution. The solution was diluted 50 times, and one drop of this solution was placed, dried on the slide and characterized by SEM (Carl Zeiss, Model, EVO 18 SEM, Germany).

#### 2.3.4 EDX study

The elemental composition and estimation of concentration of NPs of the sample was analysed by Energy-dispersive X-ray spectroscopy (EDX). The NP particle solution was sufficiently diluted, and a 10 µl of aliquots were placed on carbon stub; it was air-dried and analyzed on EDX equipped with SEM. Elemental analysis was carried out using TEAM software by taking different spots.

### 2.4 Evaluation of antibacterial activity

#### 2.4.1 Determination of MIC and MBC

The effect of various concentrations of NPs and NPs + cefixime was directed against test organisms by using diffusion assay. Different serotypes of *Salmonella* were grown on Luria and Bartuni (L.B.) agar plate at 37 °C for 24 h. The lawn growth obtained after incubation was used for antimicrobial activity analysis using the cup borer method. The small well has been created to notice a zone of inhibition of growth. Various NPs at three different concentrations and control (Antibiotic 50 µg ml^−1^) were inoculated in each separate well. Moreover, each plate was also inoculated with the lowest concentration of nanoparticles with 50 µg ml^−1^ cefixime.

## 3 Results

### 3.1 Synthesis and characterization of nanoparticles

**Fig. 1:**
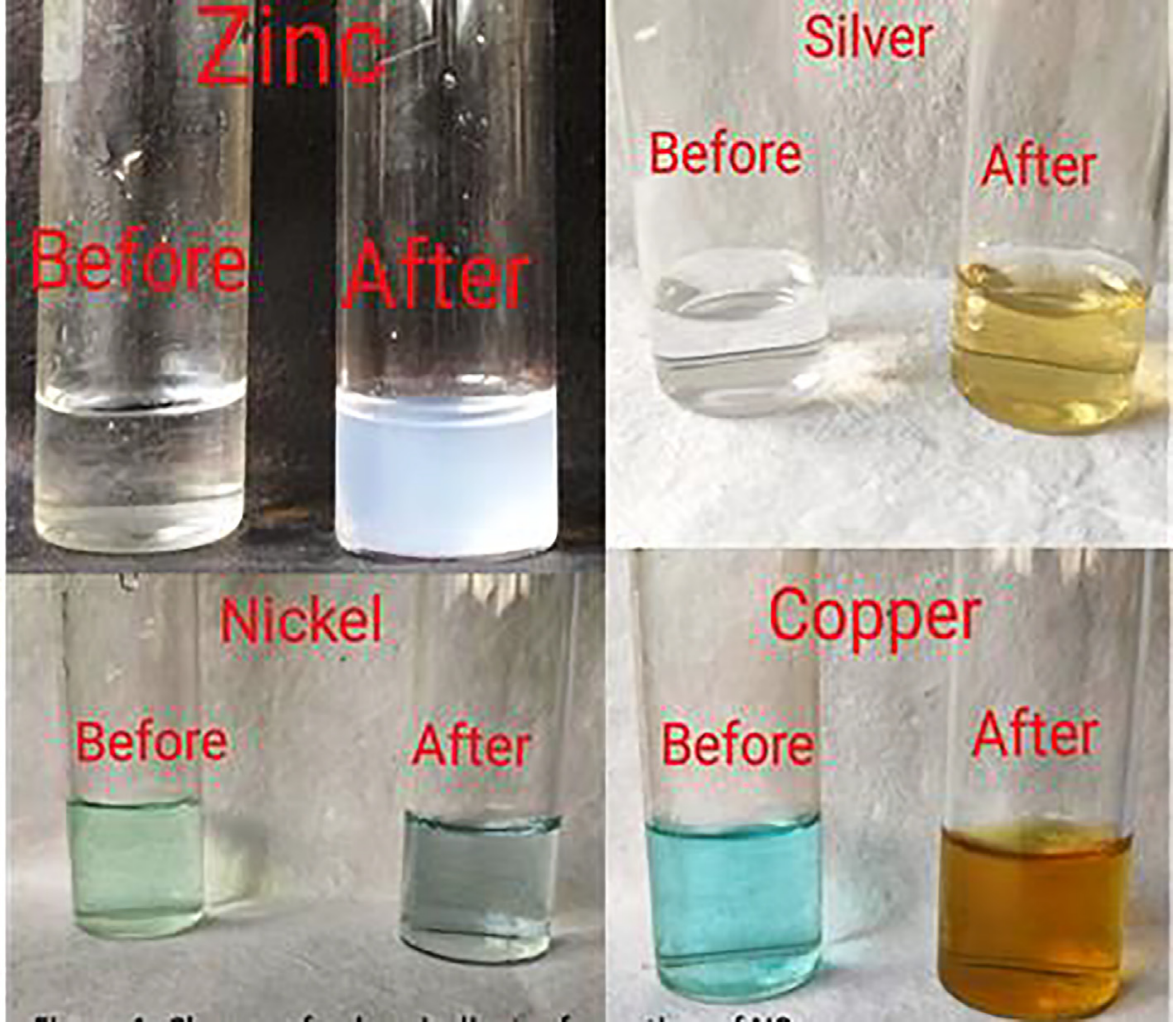
Synthesis of NPs.

During the Synthesis of NPs, a gradual change in the color development was noticed (Fig. 1).

#### 3.1.1 UV–Visible spectroscopy

**Fig. 2:**
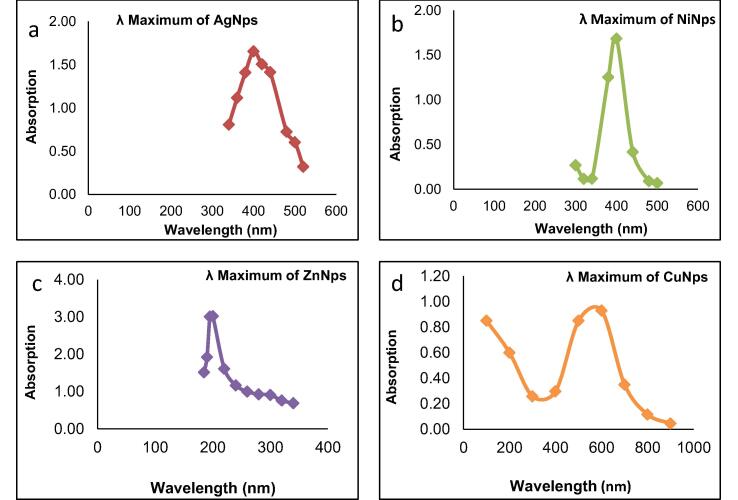
The UV–Vis spectra analysis of NPs.

UV–visible spectroscopic analysis revealed that NINp (Fig. 2a) and Agip (Fig. 2b) absorb maximally at 410 nm. This spectrum is recorded immediately after the Synthesis of particles. The Zinc nanoparticles show absorption maxima at a wavelength of 210 nm (Fig. 2c), while copper showed to be 580 nm (Fig. 2d). The absorption maxima revealed the presence of the NPs.

#### 3.1.2 FTIR analysis

All the chemically synthesized nanoparticles showed different peak intensities in FT-IR spectra according to the bond and group presents in the range 400–4000 cm^−1^. Silver NPs gave a sharp peak at 3435.48 that corresponded to O—H stretching of the alcohols and phenolic compounds. The peaks at 1638.09 cm^−1^ exhibited C=C—H stretching of the alkenes group. AgNPs exhibited a significant peak at 2063.61 cm^−1^ that reflected C=C or C≡C stretching vibrations of alkenes or alkynes. The major peak was found around 528.86 indicates metal–oxygen stretching.

Similarly, for ZnO, From the FTIR spectra, the peaks that appeared at 617 cm^−1^ in the sample are assigned to the characteristic C—H bends in alkynes. The band at 1636 cm^−1^ belonged to the stretching vibration of the N—H bond of primary amine, alkyl C=C stretch, open-chain amino group. The bend at 3423 cm^−1^ corresponded to the O.H. bond of alcohols, phenols, and primary aromatic amines. The band at 3423 cm^−1^ and 1103.71 is responsible for the stretching vibration of —O.H. bond and C—O in alkoxy. While the Cu nanoparticles showed the band at 794.67 cm^−1^, indicated a possible sp2 C.H. bend pattern of trisubstituted alkene, and rest all the values were similar as found for the other nanoparticles.

#### 3.1.3 SEM and EDAX analysis

**Fig. 3:**
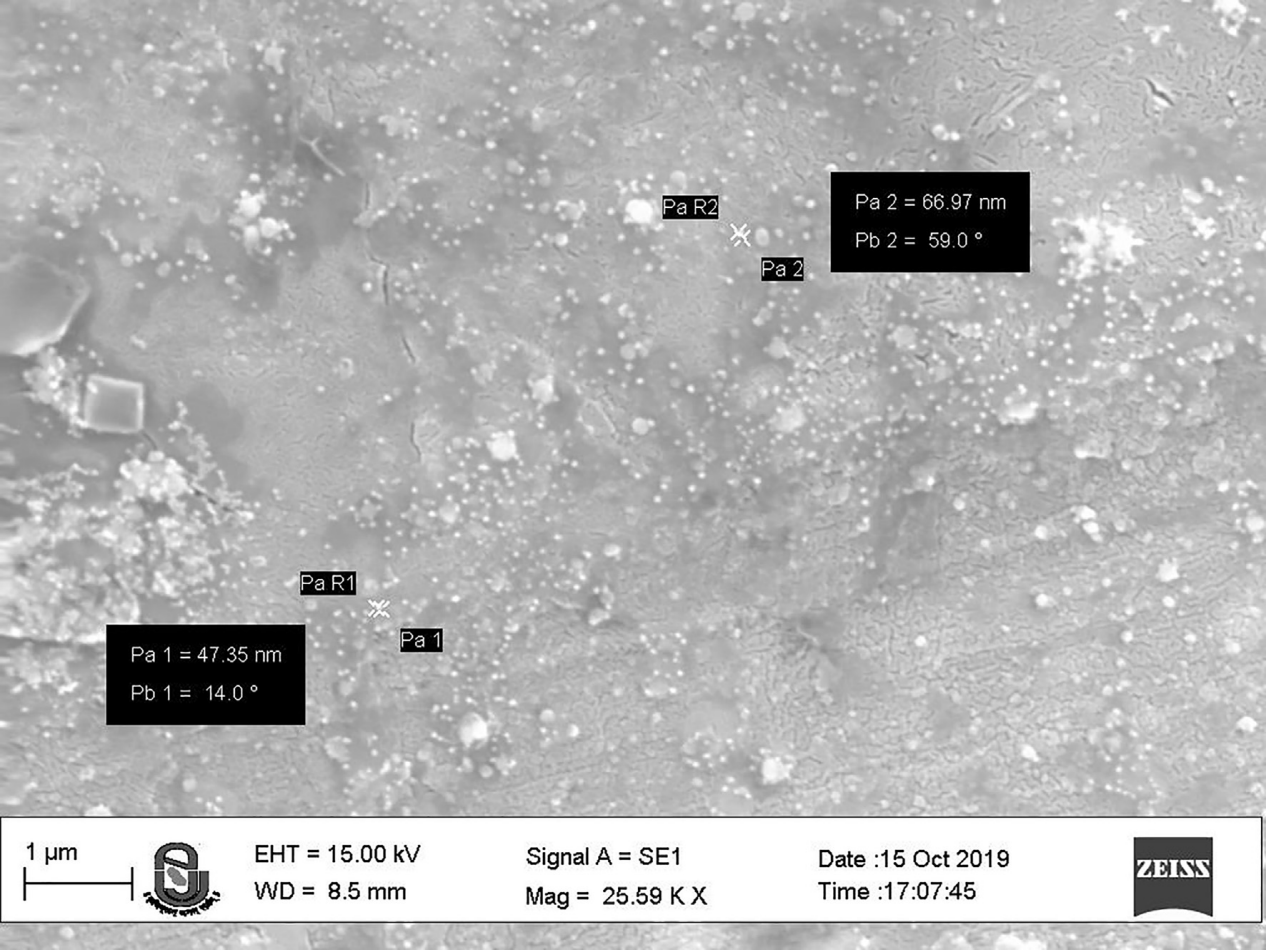
SEM image of AgNPs.

**Fig. 4:**
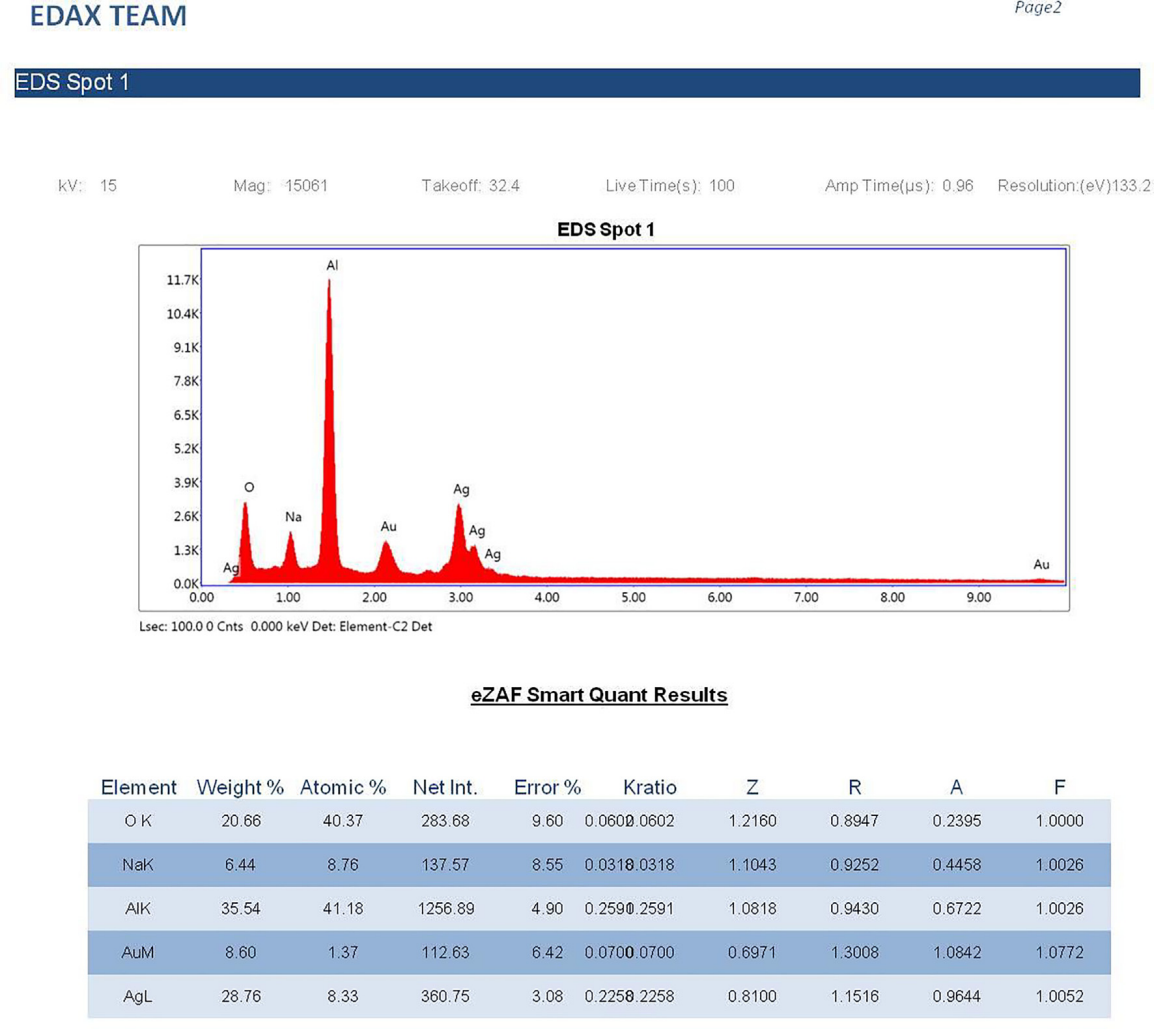

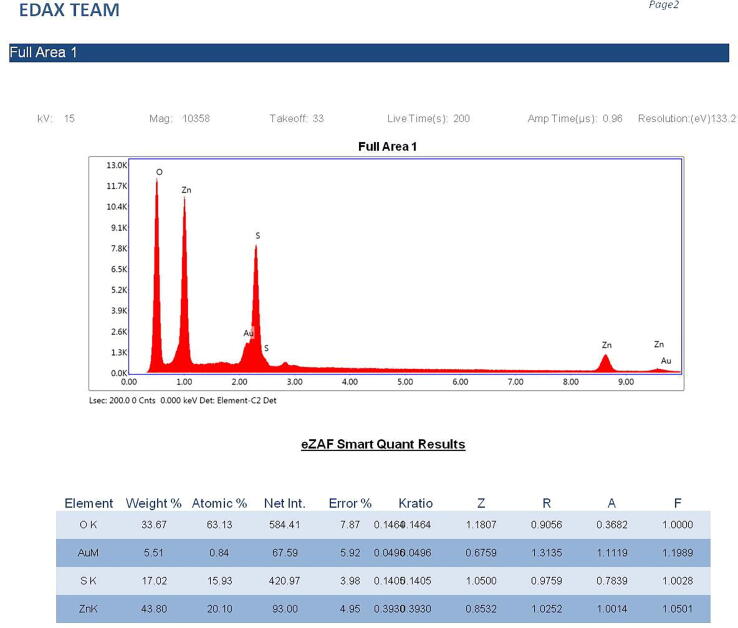

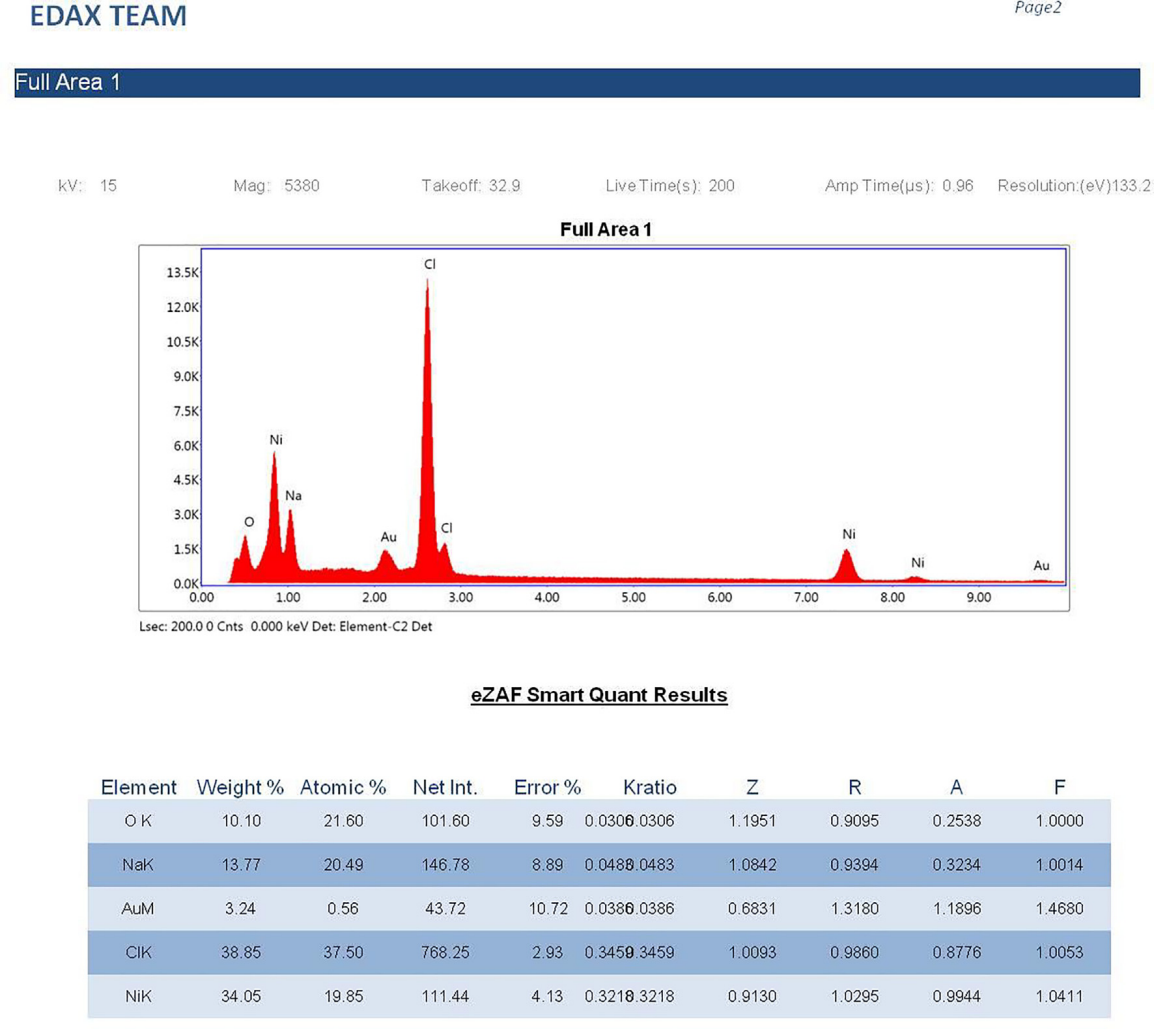

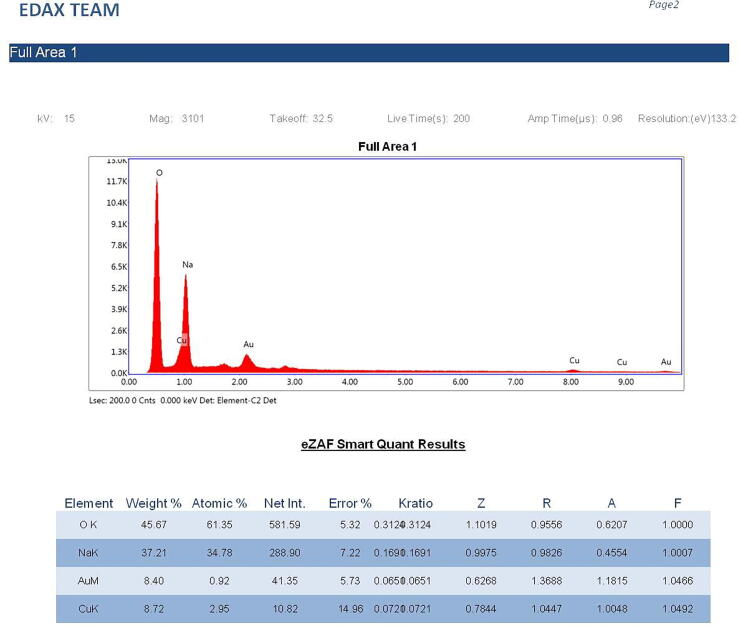
(a). Energy-dispersive X-ray analysis of AgNPs figure (b). Energy-dispersive X-ray analysis of ZnONPs. figure (c). Energy-dispersive X-ray analysis of NiNPs. figure (d). Energy-dispersive X-ray analysis of CuNPs.

The silver NPs were well dispersed, and uniform with diameter ranges between 46 nm and 66 nm (Fig. 3). The EDAX spectra of the different nanoparticles prepared using the chemical synthesis process gave the elemental composition of prepared nanoparticles indicated the presence of the respective element along with O elements. Two strong peaks correspond to the respective element and O elements that confirm the high purity of nanoparticles. The ranges of different elements used were 28.76% for silver (Fig. 4a), 43.80% for Zn (Fig. 4b), 45.49% for Ni (Fig. 4c), and 26.63 for Cu (Fig. 4d). Oxygen and carbon were the major elements found in the samples.

### 3.2 Evaluation of the antimicrobial activity of the nanoparticles

Evaluation of the antibacterial activity of various concentrations of different nanoparticles against S. typhi, S. paratyphi A, and S. paratyphi B and its comparison with the commonly used antibiotic cefixime revealed AgNP as a promising N.P. as compared to the other nanoparticles.

### 3.3 Synergistic activity of antibiotic combined with nanoparticles

The AgNPs showed a moderate zone of inhibition from 20 mm to 27 mm against the clinical isolates and the MDR strains, while the control containing the standard antibiotic exhibited the zone of inhibition from 10 mm to a maximum of 21 mm. AgNP was effective against all the strains, including the drug-resistant strains giving a higher inhibition zone than the commonly used antibiotic used as a control in the experiment. The concentration of 16.90 µg/ml of AgNp gave better results than the concentration of 50 µg in control. As the concentration of nanoparticles was increased, a higher zone of inhibition was obtained. (Table 1). The higher concentrations of NPs resulted in growth inhibition equivalent to the antibiotic commonly used. The nanoparticles were effective against the MDR strains (Table 2). NCP, CuNP, and ZnNP were ineffective against *Salmonella* strains even at higher concentrations (Table 3). A higher concentration, i.e., 3228 µg ml^−1^ of ZnNP and 498.0 µg/ml of CuNP, could not give an adequate zone of inhibition equal to the inhibition zone exhibited by the control (50 µg ml^−1^) (Table 4). However, the combination of NPs with cefixime resulted in a higher antimicrobial activity vis-a-vis NPS or antibiotics alone. The zone of growth inhibition displayed by the antibiotic was 10 mm. AgNP displayed a zone of growth inhibition of 20 mm against Salmonella typhi. The combination of antibiotics with AgNP exerted potent growth inhibition (24 mm), indicating higher efficacy of the antibiotic in the presence of nanoparticles. The antimicrobial effects of the antibiotic in combination were much better against the drug-resistant clinical isolates. The antibiotic caused less inhibition and produced 12 mm to 15 mm zone, NiNP combined with antibiotics gave inhibition of as high as 25 mm, while ZnNP with antibiotic gave 35 mm zone (Table 3) and CuNP gave as high as 38 mm zone of inhibition against the multidrug-resistant strains (Table 4).

**Table 1:** Antibacterial activity of AgNPs and antibiotics alone as well as in combination against pathogenic *Salmonella typhi.*

Test pathogens	Nanoparticles (µg)			NP + Ab (µg)	Control (µg)
	1	2	3	4	5
	16.90	25.35	33.80	(16.90 + 50)	50
	Zone of inhibition (mm)				
*S. typhi*	20	27	25	24	10
*S. paratyphi* A	20	22	20	23	10
*S. paratyphi* B	13	23	26	18	15
MDR 124	21	20	25	23	21
MDR STA_2_	25	20	27	25	15
MDR AST	20	21	22	34	23

**Table 2:** Antibacterial activity of NiNp and antibiotics alone as well as in combination against pathogenic *Salmonella typhi.*

Test pathogens	Nanoparticles (µg)			NP + Ab (µg)	Control (µg)
	1	2	3	4	5
	83.00	124.5	166.00	83.00 + 50	50
	Zone of inhibition (mm)				
*S. typhi*	11	18	20	19	21
*S. paratyphi* A	10	10	11	10	12
*S. paratyphi* B	–	–	–	14	13
MDR 124	20	22	26	22	15
MDR STA_2_	–	–	–	23	16
MDR AST	20	26	25	25	12

**Table 3:** Antibacterial activity of CuNP and antibiotics alone as well as in combination against pathogenic *Salmonella* spp.

Test pathogens	Nanoparticles (µg)			NP + Ab (µg)	Control (µg)
	1	2	3	4	5
	249	373.5	498.0	249 + 50	50
	Zone of inhibition (mm)				
*S. typhi*	15	15	20	31	22
*S. paratyphi* A	10	9	12	15	12
*S. paratyphi* B	–	–	–	12	12
MDR 124	–	9	11	20	18
MDR STA_2_	–	–	–	25	22
MDR AST	–	11	11	38	28

**Table 4:** Antibacterial activity of ZnNP as well as antibiotics alone and in combination against pathogenic *Salmonella* spp.

Test pathogens	Nanoparticles (µg))			NP + Ab	Control
	1	2	3	4	5
	1614	2421	3228	1614 + 50	50
	Zone of inhibition (mm)				
*S. typhi*	11	21	13	23	18
*S. paratyphi* A	11	20	20	17	14
*S. paratyphi* B	–	–	–	26	24-
MDR 124	–	–	–	19	18
MDR STA_2_	18	20	–	35	25
MDR AST	20	20	24	33	26

### 3.4 Determination of MIC

The Mic of the NPs were greater than 10.14 µg ml^−1^ for AgNP, 74.7 µg ml^−1^ for NiNP, 1452.6 µg ml^−1^ for ZnNP and 199.2 µg ml^−1^ for CuNP (Table 5).

**Table 5:** Minimal inhibitory concentration of various NPs against *Salmonella* spp.

NPs	*S. typhi*	*S. paratyphi* A	*S. paratyphi* B	MDR124
	Nanoparticles (µg)			
AgNP	10.14	10.14	10.14	11.83
NiNP	74.7	74.7	74.7	74.7
ZnNP	1452.6	1452.6	1452.6	1452.6
CuNP	199.2	199.2	199.2	199.2

### 3.5 Discussion

Evolution and adaptation apply to all the living forms on the earth. Microorganisms also adapt to the changing environment. The continued use of one kind of drug leads to MDR evolution in pathogens (Huh and Kwon, 2011). As a result, higher doses of antibiotics are required that cause toxicity effects. Combinatorial effect and multiple size/shape and charges of nanoparticles do not allow the resistance development in the pathogen. Thus, generalized resistance could not be developed or acquired by pathogens. The smaller nanoparticles exert more significant antimicrobial activity due to their easy diffusion in the pathogen's cell membrane (Dong et al., 2019).

The synergistic application enhances the efficiency of antibiotics towards the pathogens in a cooperative manner (Patra and Baek, 2017). The reduction in the dose of antimicrobial and low concentration of nanoparticles reduces the toxic effect on human cells and increases antimicrobial properties (Krishna et al., 2015). Naqvi et al. (2013) reported a synergistic effect of the combination of antibiotics with the silver NPs. This mixture of antibiotics and nanoparticles was highly effective against multiple drug-resistant bacteria. In synergism, the nanoparticles are surrounded by the antibiotics molecules; hence, the antibiotic concentration would be higher at the target site. The chelation effect of nanoparticles followed by antimicrobial effects leads to increased destruction of bacteria. Krishna et al. (2015) found that silver NPs, along with antibiotics like ceftriaxone and ofloxacin, enhances its efficacy significantly towards *S. typhi, S. para typhi*, and several Gram positives and Gram-negative microbes. Jyoti et al. (2016) proved the hypothesis that AgNPs along with various antibiotics could be used to kill multiple pathogenic microbes, including *Salmonella typhimurium.* They claimed good antibacterial effect of a combination of AgNps with antibiotics. We also observed similar results in our experiment that antibiotics or nanoparticles gave a lesser zone of inhibition compared to the combination. The nanoparticles significantly restricted bacterial growth. Mcshan et al. (2015) found that the potency of less effective antibiotics such as tetracycline or neomycin against multidrug-resistant *Salmonella typhimurium* DT104could is improved after combining with nanoparticles. Results of the present study also suggest that 16.90 µg AgNps along with 50 µg/ml of cefixime exerts potent antibacterial activity.

Easy penetration in the cells by small-size nanoparticle acts breaks DNA and proteins, resulting in cell lysis (Das et al., 2020). They change the membrane permeability, and the cooperative action of nanoparticles and antibiotics breaks cell walls and membranes (Kumara Swamy et al., 2015). These nanoparticles also hindered the activities of several antibiotics resistant proteins. The cell wall and nanoparticles interact due to opposite charges and augment its effects (Kumara Swamy et al., 2015).

Several scientific groups used ZnO nanoparticles and cephalosporins, beta-lactams, and aminoglycosides antibiotics to prove their antimicrobial effects against different pathogenic strains. A similar strategy was used in this study to notice the antimicrobial effect of Zn Nps (Mahamuni et al., 2019). The antibiotics, along with nanoparticles both at less but effective concentrations, inhibit the growth of microbes. Rajan et al. (2016) synthesized ZnO nanoparticles and studied their antimicrobial effects towards diverse pathogenic bacteria using the diffusion method. Joel and Badhusha (2016) obtained significant results with *Salmonella typhi* and *Klebsiella pneumonia*. Likewise, For Ni Nps also exhibited well antimicrobial activity against different bacteria and anti-fungicidal activity against *Candida albicans* (Rose et al., 2017). AgNPs improve the potency of most of the cefixime against MDR bacteria; therefore, this research provides helpful insight into typhoid treatment.

## 4 Conclusions

The continued use of a single type of antibiotic results in drug resistance in pathogens; thus, increased doses and higher concentrations of antibiotics are needed. The combination of antibiotics with nanoparticles offers aided advantages over the antibiotics or nanoparticles alone. The multidrug-resistant pathogens can be effectively killed due to a combination of a wide range of nanoparticles with cefixime or other antibiotics. Among the various nanoparticle, silver NPs exerts potent antimicrobial activity against multidrug-resistant pathogen even at the lowest concentration. Thus, this strategy would be a better alternative to combat MDR pathogens.
